# Transformational Leadership and Employee Job Satisfaction: The Mediating Role of Employee Relations Climate and the Moderating Role of Subordinate Gender

**DOI:** 10.3390/ijerph19010233

**Published:** 2021-12-26

**Authors:** Chiyin Chen, Xinyi Ding, Jiachen Li

**Affiliations:** Glorious Sun School of Business and Management, Donghua University, West Yan’an Road 1882, Shanghai 200051, China; chenchiyin@126.com (C.C.); adxy0116@163.com (X.D.)

**Keywords:** transformational leadership, employee relations climate, employee job satisfaction, subordinate gender

## Abstract

Scholars have paid extensive attention to transformational leadership for decades. However, existing studies still lack ample discussions on the underlying mechanism and boundary conditions of its influence on employee job satisfaction. This study proposed a moderated mediation model based on social exchange theory. We collected survey data from 211 frontline employees to verify our hypotheses. The results showed that transformational leadership was positively associated with employee job satisfaction via the mediation role of the perceived employee relations climate. Furthermore, the relationship between transformational leadership and the employee relations climate, as well as the indirect relationship between the two, was demonstrated to be more significant for male employees. This study offered a new account of the mechanisms of transformational leadership and clarified a boundary condition for its effectiveness.

## 1. Introduction

With the increasing competition of economic globalization and popularity of the employee-centered management approach, improving the leadership effectiveness of managers has become one of the most important ways to enhance the core competitiveness of companies and to maintain a sustainable competitive advantage [1]. In the past 20 years, transformational leadership has become one of the most popular leadership styles in both academia and in practice [2,3]. Transformational leadership is generally used to describe leaders who articulate a vision of the future that is shared with their subordinates, intellectually stimulate their subordinates, and pay attention to the individual differences among people [4]. Transformational leadership can motivate subordinates to put the interests of an organization first, as well as to put in extra effort to serve the organization [5].

Extant studies have shown that transformational leadership is positively related to the attitudes that employees have toward their jobs as well as work outcomes [6,7,8]. For example, Judge et al. [9] and Lowe et al. [10] demonstrated that there is a significant relationship between transformational leadership and the attitudes that employees have toward their jobs as well as their job performance. Employee job satisfaction refers to the attitudes or viewpoints that employees hold about their job or job experiences; as such, it is an evaluation of their overall roles at work [11,12]. The relationship between transformational leadership and employee job satisfaction is well established in the current literature [13,14,15]. Satisfied employees are a valuable organizational resource for success, well-being, and sustainability in the long run [16]. Extant studies have explored the mechanisms of transformational leadership from the perspective of intrinsic motivation and employee’s perceived relationship with the leader [17,18,19]. For example, empirical studies examined the mediation role of self-efficacy, psychological empowerment, psychological safety, and trust in leaders [20,21,22,23]. In addition to the employee’s personal motivation, job characteristics, and leader–follower interaction, recent studies on employee job satisfaction argued that employees’ perceptions of organizational settings were also critical in raising employee job satisfaction [24,25,26]. For example, Ahmad et al. [27] found that the organizational climate perceived by employees had an impact on job satisfaction. Bulińska-Stangrecka and Bagieńska [28] argued that employee relations played a role in shaping job satisfaction.

Meanwhile, research on transformational leadership has turned to discuss the impact of transformational leadership on promoting changes in the relationship between employees and the organization. Some studies argued that transformational leadership affects employee’ perceived organizational support; perceived organizational trust; and perceived climate which supports creative thinking [29,30,31,32]. However, there lacks ample empirical results to support whether transformational leadership affects employee job satisfaction from the perspective of employee’s perceived relationship with the organization. These findings raise the question of whether transformational leadership is able to affect employee attitudes toward the employee relations climate of their organization and whether this amendment in perception will affect employee job satisfaction. Empirically testing the mediating mechanism of employees’ perceived relations climate will enhance our knowledge on the effectiveness of transformational leadership. In addition, is transformational leadership effective for all employees? There is still a lack of in-depth discussion on the underlying mechanisms and boundary effects in the existing literature.

Employee relations climate refers to the shared perception and feeling of management practices among organizational members [33]. It reflects a highly engaged and employee-centered culture [33,34]. The employee relations climate is an important aspect of organizational effectiveness, and it is a source of communication between management and employees [35]. We propose that it works as a mediator between transformational leadership and employee job satisfaction. Transformational leadership will lead to close connections between employees and the organization and will create an employee-centered climate. This kind of climate will allow employees to feel the friendly side of the organization and will enable them to maintain a positive attitude in the work place, improving employee job satisfaction [36,37]. In addition, while an increasing number of gender studies on transformational leadership have explained the effectiveness of transformational leadership from the perspective of the gender of the leader, studies have ignored the gender of the subordinate [38,39]. We further argue a boundary condition of transformational leadership that is based on a subordinate gender perspective and propose that such a mediation relationship should be more significant for male employees. According to studies on gender difference, men are more achievement oriented than women at work, and they tend to pay more attention to challenges and development opportunities at work [40,41]. We contend that transformational leadership will have a more substantial effect on the employee relations climate perceived by male subordinates. Finally, we proposed and examined a moderated mediation model that incorporates the employee relations climate and gender difference into the discussion.

This study contains several contributions. First, we offer a new account to explain how transformational leadership affects employee job satisfaction from the perspective of the relationship between personal perception and the organization. It suggests that organizations can choose leaders when recruiting and can equip them with transformational leadership traits. It also reminds organizations of the importance of creating a positive employee relations climate to improve employee job satisfaction. Second, we identify how the effectiveness of transformational leadership is contingent on subordinate gender. It provides a basis for transformational leaders to classify and manage employees of different genders who are on the team. Furthermore, most research has focused on the impact of the employee relations climate at the organizational level [42,43]. Additionally, our study enriches the research on the employee relations climate by examining its individual-level effectiveness.

## 2. Literature Review

### 2.1. Transformational Leadership, Employee Job Satisfaction, and Employee Relations Climate

In organizations, climate is a measurable set of attributes of the work environment, which are perceived directly or indirectly by those who live and work in that environment [44]. The climate considers management, employees’ perceptions of how employee relations are handled, and employee interactions with each other [45,46]. The employee relations climate refers to the common perceptions that employees have about certain management practices, including interpersonal relationships, work climate, employee engagement, and performance [47]. It reflects a highly engaged, employee-centered culture [33,34]. The employee relations climate is an important aspect of organizational effectiveness [35], and current research confirms that the employee relations climate plays a mediating role between CEO relationship-focused behaviors and firm performance [42] and a mediating effect between strategy HRM practices and firm performance [43]. Establishing a positive employee relations climate is very important for an organization’s development [35]. The role of the employee relations climate between leadership style and employee attitude or behaviors deserves more attention from scholars.

Leaders play a significant role in their company and are not only the key to the company’s growth but are also the source of corporate culture [48]. Studies have shown that leaders can influence climate formation by holding a set of assumptions themselves and then by communicating them, engaging in symbolism, and inspiring consistent behaviors among their followers [49]. Additionally, the climate that is formed in the corporate environment is influenced by the leadership style. For example, Nemanich and Keller argued that transformational leaders influence subordinate outcomes through the perceived climate that they create [31,50]. Burns [51] defined transformational leadership as a behavioral process that stimulates employees to perform better at work by stimulating the spiritual aspects of their subordinates. Li and Shi [52] further combined transformational leadership with the Chinese context and identified four transformational leadership characteristics. The first characteristic, namely, be moral exemplification, suggests that transformational leaders can lead by example, consistent with their words and deeds, and demonstrate a spirit of dedication. Additionally, their morals and behaviors are recognized by employees. Second, vision motivation refers to leaders who describe the goals and visions for the company to their subordinates, allowing the employees to be more informed about the company’s future development, and the direction that they want to work toward, with the aim of bringing value to the company and to others. Third, personalized care means that leaders tend to care about the personal situations of their employees and care about their families. Finally, transformational leadership includes leadership charisma, whereby leaders have the ability to help and guide their subordinates, encouraging active innovation among their employees. Such leaders have a complete work ethic and a strong sense of professionalism, which is effective in leading employees forward.

The characteristics of transformational leadership, such as care, communication, and motivation, are beneficial in helping employees perceive a positive employee relations climate [31]. From the perspective of social exchange theory, social exchange and economic exchange in employee-organizational exchanges will have an impact on commitment and employee job satisfaction [53]. Transformational leaders are the embodiment of the organization; employees personify their organization by ascribing human-like characteristics to it [30]. Based on this organization’s personification, employees would view the organization’s expression and judge the organization’s attitude based on the leader’s behavior and attitude [54]. Transformational leaders not only convey the company’s vision and mission to employees, motivating them to work hard, but also encourage employees to innovate and challenge themselves and care about and help employees solve problems [55], which enables employees to establish a closer connection with the company. In this interactive climate, employees can feel the positive attitude and expression of the organization [36]. When transformational leaders provide help to employees when they need it, improve their abilities, impart knowledge to them, and treat them equally, employees will perceive organizational care and support. The personal and corporate interests are closely linked, and employees can perceive an employee-centered and highly involved employee relations climate, which will help improve the quality of social exchange between employees and the organization [56]. In order to fulfill the reciprocal responsibilities to the organization, employees will show more behaviors and attitudes that are beneficial to the organization as a reward for repaying or exchanging awareness [57]. When employees perceive a high-level employee relations climate, they will have positive behaviors and attitudes toward the company and contribute positive values and results to the organization, which will significantly improve employee job satisfaction. A satisfied workforce exerts more effort and works hard to achieve organizational objectives [58]. The following hypothesis is thus proposed:

**Hypothesis** **1** **(H1).** *Employees’ perceived employee relations climate mediates the relationship between transformational leadership and employee job satisfaction.*

### 2.2. The Moderating Role of Subordinate Gender

From a social information processing perspective, the effect that a leader’s behavior has on their subordinates will depend on how the subordinates perceive and process the situational cues that are conveyed by the leader [59]. Under transformational leadership, subordinates with positive characteristics are more actively engaged in their work because positive subordinates have a strong need for growth [60]. Transformational leadership develops the potential that employees have to a great extent by influencing and motivating them, thus enabling them to go beyond their interests [61]. Gender difference studies point out that men and women differ in their expectations and in their attitudes toward competition in the workplace. Generally, men value more the challenge and opportunity to develop at work. They are more willing to show their talents in competition [62]. Research has found that male subordinates pay more attention to their inner work values than female subordinates, such as being responsible and having opportunities to exert initiative and achievement [40]. Empirical studies conducted in Korea and China have supported the notion that men are more courageous, risk taking, and achievement oriented [63]. As such, they are more easily influenced by transformational leadership [64]. Our study is also rooted in a Chinese context, and we argue that the relationship between transformational leadership and perceived employee relations climate is contingent on the subordinate’s gender.

As males are more achievement oriented and value more challenge and opportunity at work, their work motivation and perceptions are more easily activated by the managerial context [65]. When transformational leaders describe the company’s future development goals to male subordinates, these male subordinates are more confident that they can bring value to the company and to others, and they feel more closely connected to the organization and are able to perceive a more positive employee relations climate [63]. However, female subordinates are relatively conservative, prefer to avoid competition, and have relatively lower expectations for developing their careers [40]. Transformational leadership creates a dilemma between the high-demand reality and the relatively conservative career expectations of female subordinates [66]. They may be less likely to prefer or embrace the values and visions of their leaders, resulting in a less positive attitude toward the organization and less engagement in their relationship with the organization [67]. Thus, the positive relationship between transformational leadership and the employee relations climate will be attenuated for female employees. Accordingly, the following hypothesis was formulated:

**Hypothesis** **2** **(H2).** *The relationship between transformational leadership and the employee relations climate perceived by employees is moderated by the subordinate gender in such a way that the above relationship is stronger for male subordinates and weaker for female subordinates.*

**Figure 1:**
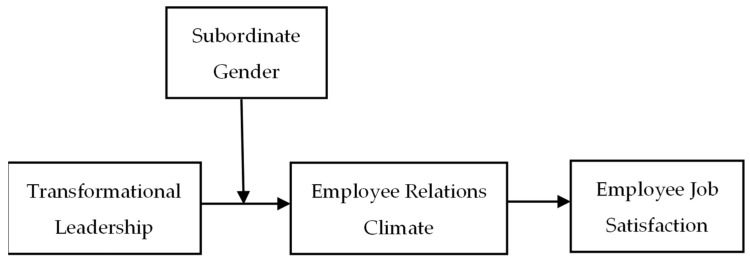
The conceptual model of the relationship between transformational leadership, employee relations climate, subordinate gender, and employee job satisfaction.

Integrating the mediating role of Hypothesis 1 and the moderating role of Hypothesis 2, we further proposed an integrated moderated mediation model in which the mediating effect of the perceived employee relations climate is moderated by subordinate’s gender. Transformational leadership emphasizes interaction and connection between leaders and subordinates [66], which has a direct impact on the employee relations climate [46], and the employee’s perception of this climate has a positive impact on employee job satisfaction. In addition, the subordinate’s gender can play a moderating role in the positive role that transformational leadership plays in employee job satisfaction through the perceived employee relations climate. Specifically, male subordinates attach importance to development opportunities and job challenges, and they tend to benefit more from transformational leaders who convey their goals and visions of the company to them [65]. When employees have a stronger perception of an employee-centered climate and support from the organization, they will have more positive perceptions of the relations climate in the organization, resulting in enhanced employee job satisfaction [63]. Conversely, female subordinates are relatively conservative and more inclined to avoid competition [40]. They are lower in achievement orientation and may be less sensitive to the values and visions of their leaders, resulting in fewer perceptions about the employee relations climate, resulting in a lower level of employee job satisfaction [66]. Accordingly, the following hypothesis was formulated, and Figure 1 illustrates the conceptual framework.

**Hypothesis** **3** **(H3).** *The indirect relationship between transformational leaders and employee job satisfaction via employees’ perceived employee relations climate is moderated by the subordinate’s gender. Specifically, this indirect relationship is stronger for male subordinates and weaker for female subordinates.*

## 3. Methods

### 3.1. Participants and Procedure

We use survey data to examine our hypotheses. To ensure the anonymity of the research subjects and to allow them to express their opinions truthfully, data for this study were collected from various industries using an online survey platform called WJX.cn [68]. The platform is a third-party platform that follows very strict sample collection procedures to ensure valid responses, and it provides services that can be used to collect data from the target population. We recruited participants to answer our questionnaire via this platform. According to the ratio criteria (one item needs five responses), we planned to recruit around 250 participants for the survey. After the survey had been posted for one week, we received a total of 248 responses from frontline employees in China and eliminated 37 due to incomplete answers and missing information. This study is a cross-sectional design study. The final number of usable responses was 211, providing an effective recovery rate of 85.1.

Table 1 shows the characteristics of the respondents. In terms of the sex ratio of the respondents, 40.76% were male and 59.24% were female, which reflected the gender balance of the sample. In terms of age, the average age of the participants was 33.06 years old, and the majority of the respondents were 20–29 years old. From the perspective of educational background, tenure, and company industry, the participants were properly distributed for each aspect.

### 3.2. Measures

Unless otherwise indicated, the measures used five-point Likert scales ranging from 1 “strongly disagree” to 5 “strongly agree”.

Transformational leadership (TL): We used the twenty-six-item scale determining transformational leadership that was proposed by Li and Shi [52]. An example item from this scale is “My leader endures hardship first and enjoys last”. We averaged the twenty-six-item scores to create a total scale score (Cronbach’s α = 0.912, mean = 4.113, SD = 0.373).

Employee relations climate (ERC). We used the eight-item scale to determine the perceived employee relations climate that was developed by Ngo et al. [47]. An example item from this scale is “I can fully utilize my knowledge and skills in the organization”. We averaged the eight-item scores to create a total scale score (Cronbach’s α = 0.714, mean = 4.118, SD = 0.455).

Employee job satisfaction (EJS). We used the twenty-item scale measuring employee job satisfaction that was created by Weiss [69]. An example item from this scale is “I have the opportunity to work independently”. We averaged the twenty-item scores to create a total scale score (Cronbach’s α = 0.875, mean = 4.061, SD = 0.369).

Subordinate gender. We controlled for subordinate gender (0 = male, 1 = female).

Control variables. We controlled for age, education background (1 = high school or below, 2 = bachelor’s degree, 3 = master’s degree), company’s industry (0 = manufacturing, 1 = service industry and others), and working years.

### 3.3. Analysis Strategy

First, we employed Harman monofactor analysis to analyze the common method biases and CFA analysis to assess the measurement validity. Second, we conducted a correlation analysis. Then, we employed ordinary least squares (OLS) analysis in Mplus 7.0 (Muthén & Muthén, Los Angeles, CA, USA) to test the hypothesis. We further applied the Monte Carlo approach to examine the indirect effect. Then, we conducted a simple slope test to examine the moderation effect. Finally, we conducted the Monte Carlo approach to test the moderated mediation hypothesis.

## 4. Results

### 4.1. Measurement Validation

As the survey was a self-evaluation for employees, we used the Harman monofactor analysis to analyze the common method biases of the sample data [70]. The results show that the unrotated monofactor interpretation variable was 25.88%, which did not account for half of the total variance that was explained. Additionally, after the data for each variable were centralized, the tolerance range was 0.90–0.98, and the variance inflation factor was less than 2.0. Therefore, it could be determined that there were no severe multicollinearity problems between the variables.

A confirmatory factor analysis (CFA) was performed to assess the measurement validity. We subjected the three sub-constructs for the TL, ERC, and EJS to one CFA. As TL contains many levels and because EJS contains a large number of items, we used the item-parceling strategy to improve the model fitness [71,72]. We parceled TL according to the four dimensions and EJS according to factor loading. The results showed that the three-factor model fit the data well (χ^2^ = 132, df = 88, *p* < 0.01, RMSEA = 0.0487, CFI = 0.969, TLI = 0.963). This baseline model was significantly better than the other two-factor models. The first two-factor model combined employee relations climate and employee job satisfaction into one factor (χ^2^ = 140, df = 89, ∆χ^2^ = 8, ∆df = 1, *p* < 0.01), the second two-factor model combined transformational leadership and employee job satisfaction into one factor (χ^2^ = 246, df = 90, ∆χ^2^ = 114, ∆df = 2, *p* < 0.01), and the third two-factor model combined transformational leadership and employee relations climate into one factor (χ^2^ = 181, df = 89, ∆χ^2^ = 49, ∆df = 1, *p* < 0.01). Additionally, this baseline model was also significantly better than the single-factor model (χ^2^ =246, df = 90, ∆χ^2^ =114, ∆df = 2, *p* < 0.01). Overall, the discriminant validity of the constructs was confirmed.

### 4.2. Correlation Analyses

Table 2 shows the correlations and reliabilities of each variable. A significant positive correlation was observed between transformational leadership and the employee relations climate and between transformational leadership and employee job satisfaction. At the same time, there was also a significant positive correlation between the employee relations climate and employee job satisfaction. The relationship between the variables was in line with the expectations of the study.

### 4.3. Hypothesis Tests

We employed OLS in Mplus 7.0 to test our hypotheses. The regression results are shown in Table 3. Model 1 regressed the effect of employee job satisfaction (EJS) on transformational leadership (TL). Model 2 regressed the effect of EJS on TL and the employee relations climate (ERC) simultaneously. Model 3 regressed the effect of ERC on TL, gender, and their interaction term. Moreover, Model 4 regressed the effect of EJS on TL, gender, the interaction term of TL and gender, and ERC simultaneously.

H1 argued that the employee relations climate mediated the relationship between transformational leadership and employee job satisfaction. Incorporating the results of Model 1 and Model 2, we can observe that the positive relationship between transformational leadership and employee job satisfaction remained significant after adding the employee relations climate into the regression model. However, the coefficient decreased significantly, indicating that the employee relations climate played a partially intermediate role between transformational leadership and employee job satisfaction. To examine the indirect effects, we applied the Monte Carlo approach to generate the confidence intervals (CIs) [73]. We constructed bias-corrected 95% CIs for the indirect effects based on 2000 re-samples. Bootstrap analysis showed that the mediating effect of transformational leadership on employee job satisfaction via employee relations climate was significant (indirect effect = 0.392, CI = [0.294, 0.514], not containing 0). Therefore, H1 was further supported.

**Figure 2:**
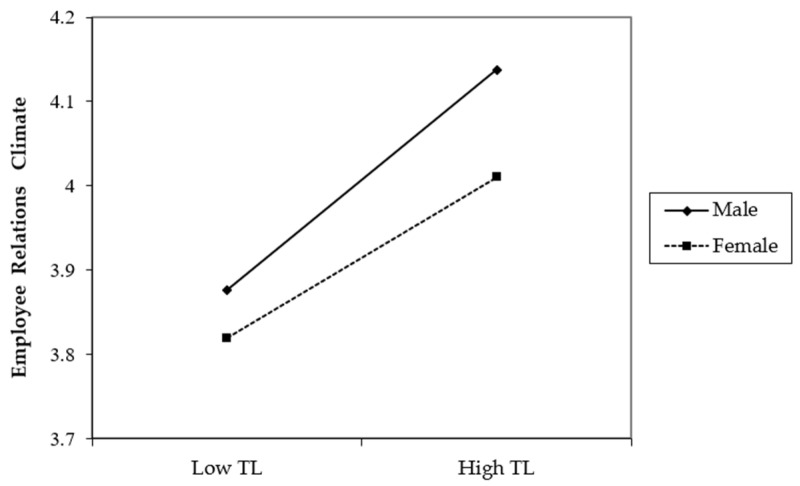
The influence of transformational leadership on employee relations climate under different subordinate genders.

We tested H2, which considered whether subordinate gender played a moderating role between transformational leadership and the employee relations climate. As shown in Table 3, the interaction term between transformational leadership and subordinate gender had a significant effect on the employee relations climate in Model 3. To examine the moderating role of subordinate gender more visually, we plotted the moderating role of the employee relations climate in Figure 2 and conducted a simple slope test. Figure 2 suggests that the positive effect of transformational leadership on the employee relations climate was more significant for male subordinates (b = 0.394, *p* < 0.01). In contrast, the effect of transformational leadership on the employee relations climate was relatively weaker when the subordinate’s gender was female (b = 0.308, *p* < 0.01), and the difference between the two conditions was significant (b = −0.086, *p* < 0.05). Therefore, H2 was supported.

We further examined the moderated mediation model in which the subordinate gender should moderate the indirect effect. The results of Model 4 in Table 3 show that the interaction term between transformational leadership and subordinate gender was no longer significant but that the employee relations climate predicted employee job satisfaction. The bootstrap analysis results in Table 4 showed that the mediation effect of transformational leadership on employee job satisfaction via the employee relations climate was significant when the subordinates were male; when the subordinates were female, the above relationship remained significant. Additionally, the difference was significant, and the indirect effect was stronger for male subordinates. Therefore, H3 was supported.

## 5. Discussion

### 5.1. Theoretical Contributions

This study offers several theoretical contributions. First, our study reveals a new mechanism of transformational leadership. Previous studies have verified the relationship between transformational leadership and employee job satisfaction from intrinsic motivation and trust in leaders [17,18,19]. Recent studies found that the employee’s job satisfaction will also be affected by the perceived organizational interactions [24,25,26]. Therefore, we offer a new account from the perspective of employees’ perceived relationship with the organization to discuss the effect of transformational leadership on employee job satisfaction. Specifically, we examined the mediator of perceived employee relations climate. Transformational leadership emphasizes the connection and interaction between leaders and employees. The behavior of transformational leaders affects the formation of the employee relations climate within the company, which will further influence employee job satisfaction after employees perceive a positive employee relations climate. Our empirical conclusions are consistent with those of the previously published literature on employee job satisfaction, which indicates that the organizational climate is positively related to employee job satisfaction [27].

Second, this study contributes to the literature on the boundary conditions of transformational leadership based on a subordinate gender perspective. Studies of gender differences in transformational leadership focus on leaders’ gender [38,39]. However, studying whether and why transformational leadership produces different effects between male and female subordinates is also examined in the literature [74]. In previous studies, subordinate gender was generally only used as a control variable [63]. This study empirically regarded subordinate gender as the moderator variable and found that subordinate gender moderated the relationship between transformational leadership and the employee relations climate as it was perceived by the employees, in turn, affecting employee job satisfaction. Therefore, this study expands the gender theory as it pertains to the effectiveness of transformational leadership. In the future, research on the subordinate gender is worthy of attention in transformational leadership.

Last but not least, our study enriches the research on employee relations climate by examining its individual-level effectiveness. Existing research has focused on the impact of the employee relations climate at the organizational level. For example, research found that the employee relations climate had a positive impact on organizational performance [42,43]. We further empirically found that the employee relations climate mediated the relationship between transformational leadership and employee job satisfaction. In addition, subordinate gender played a moderating role in the mediation of the employee relations climate between transformational leadership and employee job satisfaction. Our results provide insight into how the employee relations climate has a positive impact on individual outcomes.

### 5.2. Management Implications

The present research shows how employee job satisfaction can be effectively improved through transformational leadership behavior. The following implications can be drawn from the present research:

First, we suggest that organizations choose supervisors with transformational leadership traits when recruiting. Because transformational leadership can effectively predict employee job satisfaction [13,14], the organization can add relevant questions during the recruitment test and interview to examine the leader’s moral qualities, their level of concern for their employees, and the candidate’s ability to plan goals. For managers who do not have these traits, it is essential to organize training to help these managers acquire transformational leadership skills, such as how to help employees at work and in life, improve their capabilities and leadership charm, and strengthen their interactions with their employees.

Second, organizations should carry out training programs to improve manager awareness and their ability to establish an employee relations climate that is perceived as positive by their employees, as climate mediates the relationship between transformational leadership and employee job satisfaction. Bowen and Ostroff argued that organizational intangible resources, such as organizational climate, create sustainable competitive advantages for the company; therefore, it is important to manage them properly [37]. This paper reminds organizations to put more effort into creating a positive employee relations climate. Transformational leadership as the incarnation of an organization has an important influence on forming the social climate [30]. They need to promote the formation of a positive employee relations climate and consciously enhance employees’ perception of the employee relations climate.

Finally, transformational leaders should notice that male employees and female employees perceive the effectiveness of transformational leadership differently. Previous research showed that the impact of transformational leadership on employee job satisfaction varied according to employees’ individual characteristics, such as education background [75]. Our study finds that subordinate gender also plays a moderating role in the efforts taken by transformational leaders to improve employee job satisfaction through the perceived employee relations climate. Specifically, this relationship is more pronounced for male subordinates. Therefore, the desire to improve employee job satisfaction by establishing a positive employee relations climate is more evident in organizations with more male subordinates.

### 5.3. Limitations and Further Research

Our study has some limitations, and these should be considered in future research. First, all of the hypotheses were tested using cross-sectional data, which did not allow accurate conclusions about the causal relationships between variables. We encourage future researchers to use a longitudinal design to examine the causal relationships between transformational leadership and employee job satisfaction. We also recommend that researchers use a mixed method, for example, conduct interviews and use field samples. Second, this article studies the concept of transformational leadership as a whole, but transformational leadership consists of multiple dimensions. Future research should continue to investigate how different dimensions under transformational leadership affect employee job satisfaction. Third, this study examined the role of the employee relations climate as perceived by employees at the individual level. Future research could study the impact of the employee relations climate at the team level.

## 6. Conclusions

This study provided a theoretical model of transformational leadership, the perceived employee relations climate, subordinate gender, and employee job satisfaction. The results showed that the perceived employee relations climate partially mediated the relationship between transformational leadership and employee job satisfaction. Subordinate gender moderated the relationship between transformational leadership and the perceived employee relations climate. In addition, the indirect relationship between transformational leaders and employee job satisfaction via perceived employee relations climate was moderated by subordinate gender, and this indirect relationship was stronger for male subordinates. This study offers a new account of the mechanisms of transformational leadership and clarifies a boundary condition for its effectiveness.

## Figures and Tables

**Table 1:** Characteristics of the respondents.

Characteristics		Frequency	Percentage (%)
Gender (mean = 0.590, sd = 0.493)	Male	86	40.76%
	Female	125	59.24%
Age (mean = 33.060, sd = 12.824)	20–29	110	52.13%
	30–39	38	18.01%
	40–49	18	8.53%
	>50	45	21.33%
Education (mean = 1.900, sd = 0.589)	High school or below	48	22.75%
	Bachelor’s degree	136	64.45%
	Master’s degree	27	12.80%
Tenure (mean = 10.060, sd = 4.113)	<3	64	30.33%
	3–6	51	24.17%
	7–10	23	10.90%
	>10	73	34.60%
Company’s industry	Manufacturing	28	13.27%
	Service and others	183	86.73%
	Financial service	40	18.96%
	Real estate	13	6.16%
	Education	25	11.85%
	Internet	22	10.42%
	Wholesale and retail	25	11.85%
	Public administration	16	7.58%
	Others	42	19.91%

**Table 2:** Correlations and reliabilities.

Variables	Gender	Age	Education	Industry	Tenure	TL	ERC	EJS
1. Gender								
2. Age	−0.111							
3. Education	−0.042	0.561 **						
4. Industry	0.125	−0.014	−0.029					
5. Tenure	0.135	0.933 **	0.609 **	0.023				
6. TL	−0.128	0.019	0.083	−0.092	−0.015	***(0.912)***		
7. ERC	−0.113	0.293 **	−0.089	0.047	0.282 **	0.644 **	***(0.714)***	
8. EJS	0.154 **	0.140 *	0.027	0.010	0.157 **	0.628 ***	0.747***	***(0.875)***

Note: * *p* < 0.05, ** *p* < 0.01, *** *p* < 0.001 (two tailed). Italic and bold numbers in parentheses represent the variable reliability. TL = transformational leadership; ERC = employee relations climate; EJS = employee job satisfaction.

**Table 3:** Hierarchical multiple regression of employee relations climate and employee job satisfaction.

	EJS	EJS	ERC	EJS
	Model 1	Model 2	Model 3	Model 4
Intercepts	1.379	1.208	3.659	2.235
Age	0.000	−0.005	0.010	−0.005
Education	0.023	0.003	0.048	−0.001
Industry	0.070	−0.004	0.162 *	0.010
Tenure	0.006	0.005	0.003	0.005
Transformational leadership	0.627 **	0.233 **	0.350 **	0.110 **
Subordinate gender			0.001	−0.042
Employee relations climate		0.489 **		0.479 **
TL × Subordinate gender			−0.094 *	−0.040
R^2^	0.428	0.599	0.533	0.604

Note: * *p* < 0.05, ** *p* < 0.01. TL = transformational leadership. The results of the standardized regression coefficients.

**Table 4:** The moderated mediation model test.

	95% Confidence Level (CI)		
Subordinate Gender	Indirect Effect	Boot LCI	Boot UCI
Male	0.189	0.131	0.261
Female	0.147	0.112	0.191
Variance	−0.041	−0.080	−0.006

Note: Bootstrapping sample size = 2000.

## Data Availability

The data presented in this study are available on request from the corresponding author.
